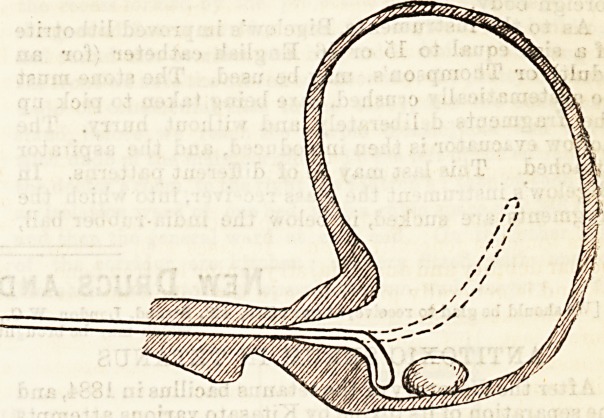# The Treatment of Stone in the Bladder

**Published:** 1893-08-12

**Authors:** 


### BRISTOL ROYAL INFIRMARY.

### The Treatment of Stone in the Bladder.

It is almost unnecessary to state that the success of an operation largely depends on the care bestowed on details. This is very noticeable in the class of cases dealt with in this paper; and the object of the following remarks is to point out some of the minor points which should be attended to. The main steps of a modern operation for stone do not differ much in different hospitals, and about these little need therefore be said.

No institution, perhaps, has a longer record of lithotomies and lithotrities than the Bristol Royal Infirmary, and the remarkable collection of calculi in its museum bears witness to the skill and audacity of former surgeons. We may therefore briefly indicate a few points in the traditions and practice of this institution as a type of the general routine in this country.

First, as to sounding. Occasional mistakes are inevitable; for the grating produced by phosphatic deposit, especially if this be on the surface of a growth, almost exactly simulates the rough sensation caused by a calculus. It is a known fact that Dupuytren on one occasion at least operated, and found nothing in the bladder. The calculus, again, may escape detection from its small size or from its position. The "sound" generally used is Sir Henry Thompson's, with a short, sharply curved extremity. A glance at the diagram will show how much easier it is to detect a foreign body with such an instrument, than with the larger curved one represented in dotted outline. The danger of making a mistake as to the presence of a stone, led to the rule at one time strictly observed at the Bristol Infirmary, viz., that no cutting operation should in any case be performed unless *all* the surgeons present at the consultation could detect the existence of a stone. Another point of importance is the amount of fluid in the bladder. If this organ is enlarged from over distension a calculus might easily be missed, and if it be too empty the corrugations of the mucous membrane are apt to get in the way of the sound. Moderate distension with urine or with warm boric acid solution is desirable.

*Lithotomy*, either perinæal or supra-pubic, is necessary if the stone is too hard or too large for crushing. No definite rules are possible. The age of the patient, the condition of the bladder and other organs, and especially the hardness of the calculus, are all to be considered. Very large stones are, however, seldom met with now, probably because relief is sought earlier. Oxalate of lime and uric acid stones of more than one inch diameter can seldom be crushed.

The *bowels* must be cleared by a simple enema before the operation, and the bladder should be emptied and refilled with a warm saturated solution of boric acid. Much depends on the manner in which the *staff* is held, and this is so important that this part of the operation is supposed to be given to the senior surgeon present. Steadiness and firmness are especially requisite.

The *incision* should be as free as is consistent with the safety of the deeper structures. Nothing is gained by too short a cut, and extraction may be made thereby an unnecessary laceration, and the bladder has even been separated from the urethra in some unfortunate cases.

The irrigator should be used after the operation, and the parts well flushed with an antiseptic lotion.

Drainage tubes are not generally employed.

The patient should be kept warm, and every precaution taken (by the use of absorbent and antiseptic wool) to prevent the urine getting about. A mild morphia draught may be necessary.

The operation of *Lithotrity* has been revolutionised since Bigelow introduced the "evacuator" in 1878. A few years ago a great authority cautioned the profession in a most impressive manner against keeping a lithotrite in the bladder for more than a minute or two at a "sitting." Now, calculi of over three ounces in weight are removed by crushing at operations that have lasted for hours.

It may be noted that a moderate amount of cystitis does not preclude the operation, but it is important to get rid of this before the crushing, if possible. For this purpose a few days' absolute rest in bed, with careful washing out the bladder twice daily, is very effectual. Cystitis, even in severe cases, being dependent chiefly on decomposition of the urine, not on irritation by the foreign body.

As to the instruments, Bigelow's improved lithotrite of a size equal to 15 or 16 English catheter (for an adult), or Thompson's. may be used. The stone must be systematically crushed, care being taken to pick up the fragments deliberately and without hurry. The hollow evacuator is then introduced, and the aspirator attached. This last may be of different patterns. In Bigelow's instrument the glass receiver, into which the fragments are sucked, is below the india-rubber ball, and it is not so easy to see the return current with its particles of stone as in Thompson's aspirator. In this there is no possibility of pieces of calculus getting into any part of the apparatus except the glass reservoir, and an excellent view is obtained of the fluid as it comes from the bladder.

The pain following the operation is generally inconsiderable, but if it should be severe a dose of henbane and morphia may be given, and sponge cloths rung out in water as hot as the patient can bear it can be placed on the perinæum and lower part of the abdomen. It is not usual to allow the patient to get up before the fifth or sixth day at the earliest.

## Figures and Tables

**Figure f1:**